# Supplementary material for: 150 MHz polymer resonator for optoacoustic mesoscopy based on a tapered optical fiber
Source: Nat Commun. 2026 May 12;17:4328. doi: 10.1038/s41467-026-72815-9 (PMC13172559; doi:10.1038/s41467-026-72815-9)
Supplement: Supplementary file 1 — Supplementary Information [file 41467_2026_72815_MOESM1_ESM.pdf]

Supplementary Material

TOF-PR angled-polishing procedure

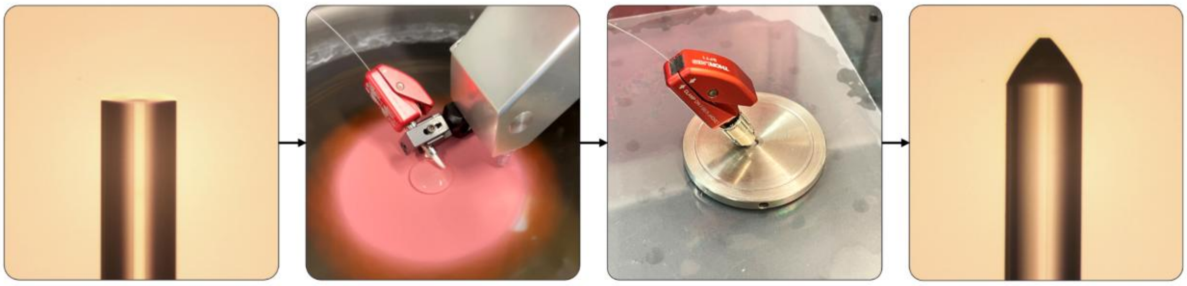

Supplementary Figure 1. Angled-polishing procedure to create tapered optical fibers. The optical fiber is initially flat-cleaved. A motorized fiber polishing machine tapers the fiber tip, creating a conical shape. Hand polishing with a custom-made disc further refines the tip, reducing surface irregularities and ensuring a symmetrical cone, which is verified with inspection under a microscope. The fiber tip is then flat polished with a standard disc (not shown) to achieve the desired base diameter for the polymer resonator to be built in the next step. Adapted from an image by the author (O. Ülgen) originally published in the thesis "Miniaturized Optical Ultrasound Detectors for High-Resolution Optoacoustic Imaging", Technical University of Munich, 2023 [1].

The manufacturing process starts with the flat cleaving of the optical fiber, followed by angle-polishing, as detailed in **Suppl. Fig.1**. This technique created a tapered fiber tip, which served as the substrate for the polymer resonator (PR). To facilitate angle-polishing, we modified a commercially available optical fiber termination latch to allow the fiber to rotate around its axis during the polishing process. Initially, the latch was attached to a holder on a motorized fiber polishing device. Next, a diamond polishing sheet with a finer grit size (0.01 µm) and a custom-made disc were used for manual polishing. To achieve a symmetrical conical tip profile, the fiber was rotated at a constant speed around its axis within the polishing ferrule while maintaining a steady 30° tilt from the normal axis of the polishing paper. This consistent tilt was maintained by the custom-made disc, which could be redesigned to alter tilt angle. After polishing, the fiber was cleaned using sonication and isopropyl alcohol. Finally, the PR was formed using UV-curable epoxy, and silver mirrors were deposited through dip-coating.

Optoacoustic micro-tomography setup

Suppl. Fig.2a shows the all-optical raster-scanning optoacoustic micro-tomography setup used for imaging experiments. The optoacoustic excitation source (Innolight, Germany, Flare PQ HP GR 2k-500) generated 1 ns-pulses with a wavelength of 515 nm and a pulse repetition rate of 1.2 kHz. The fiber-based collimator (F810SMA-543, Thorlabs) coupled the excitation light into a 200 µm-core multi-mode optical fiber (M92L02, Thorlabs) which then delivered light onto the sample over an area of approximately 1 mm^2. The detection fiber (**Suppl. Fig.2b**) was positioned above the illumination area using motorized translation stages (MTS50/M-Z8, Thorlabs). The signal acquisition began when an Si photodiode (DET36A, Thorlabs) detected excitation pulses in free space.

For optical interrogation, we used a continuous-wave tunable sweep laser (Intun TLX-1550B, Thorlabs) **(Suppl. Fig. 2)**, The wavelength of the read-out beam was set to the optical resonance quadrature point **(Fig. 1b)**. To complete the read-out loop, a fiber-based circulator (CIR1550PM-APC, Thorlabs, Germany) directed the interrogation beam through the resonator and routed the reflected light to a photodiode (PDB480C, Thorlabs, Germany). The resulting signals were digitized at a sampling rate of 3 GS/s (CSE123G2, GaGe; Lockport, USA) and stored for further analysis. During the acquisition, the mean amplitude (DC-bias) of the reflected signal was continuously monitored to ensure that the read-out beam remained precisely tuned to the quadrature point of the detector.

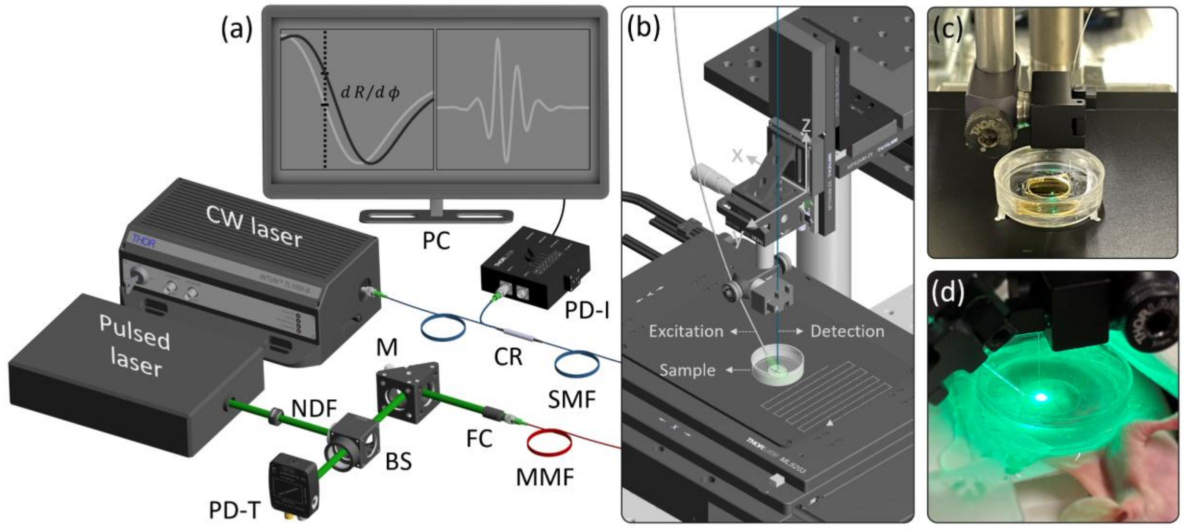

Supplementary Figure 2. All-optical raster-scanning optoacoustic micro-tomography setup. **(a)** The main components of optical delivery and signal acquisition. An optoacoustic excitation source generates ns-pulsed laser beams of 532 nm. The excitation light is coupled into a multi-mode optical fiber using a fiber-based collimator and delivered onto samples. The continuous-wave (CW) interrogation laser is connectorized to the fiber probe using polarization-maintaining (PM) single-mode optical fiber (SMF) and a fiber-based coupler. The optical power of the excitation pulses is attenuated using neutral density filters. The beam-splitter positioned next to the excitation source redirects a small portion of light onto a photodiode which triggers signal acquisition. Abbreviations: BS: Beam-splitter, CR: Circulator, FC: Fiber collimator, M: Mirror, MMF: Multi-mode fiber, NDF: Neutral density filter, PD-I: Interrogation photodiode, PD-T: Trigger photodiode, SMF: Single-mode fiber. **(b)** The raster-scanning system includes top stages for the alignment of the imaging probe in xyz coordinates and the microscopic stages for the translational scanning. **(c)** Experimental setup with a thin (200nm) gold plate for the spatio-temporal characterization of the optical fiber-based detectors. **(d)** Experimental setup for in-vivo optoacoustic imaging of back skin of mouse. Adapted from images by the author (O. Ülgen) originally published in the thesis "Miniaturized Optical Ultrasound Detectors for High-Resolution Optoacoustic Imaging", Technical University of Munich, 2023 [1].

Bandwidth characterization of PRs

The spatio-temporal characteristics of the developed optical fiber-based detectors were determined using a 200-nm-thin gold plate **(Suppl. Fig.2c)**. A diffraction-limited optical focal spot was used to create an ultra-wideband optoacoustic point source. In this characterization setup, the fiber-based detector captured the optoacoustic signals in transmission mode. The acquired signals were averaged 1000 times and digitally filtered to 2-500 MHz in the post-processing. For bandwidth analysis, the recorded signal was transformed to the frequency domain using fast Fourier transform.

Volumetric optoacoustic imaging

The artifact-free imaging capabilities of the optical fiber-based detector facilitate the acquisition of three-dimensional optoacoustic images of the intricate vascular system without the loss of information. **Suppl. Fig.3** depicts the volumetric optoacoustic reconstruction of a 2 mm x 2 mm area scanned with a 5 µm step size. The reconstructed section has a thickness of 370 µm across the working distance from 480 µm to 850 µm. We took measures to maintain a low fluence level that is sufficient to generate optoacoustic signals throughout the entire thickness of the sample. As the tissue depth increased, the amplitude of the optoacoustic signals produced decreased because of the scattering and absorption of light within the tissue. The detector's ability to capture fine details at depths greater than 1 mm was demonstrated in this experiment. Through our investigation and analysis of raw signals obtained with the PR on a tapered optical fiber, we concluded that the impact of surface acoustic waves (SAWs) on tomographic image reconstruction was negligible. Our empirical results clearly show that the developed detector can produce high-resolution volumetric optoacoustic images of complex structures, effectively mitigating the negative effects of SAWs.

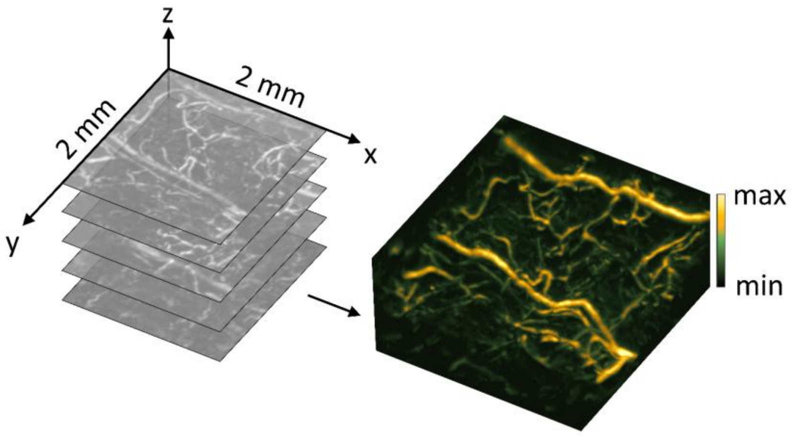

Supplementary Figure 3. Volumetric maximum intensity projection of mouse ear vasculature in vivo. The reconstructed image shows a 2 mm x 2 mm area scanned with a 5 µm step size. The volumetric image is reconstructed for the depth range from 480 µm to 850 µm, which is rich in vasculature. The fiber probe provides high-contrast imaging down to a depth of 850 µm, allowing for the clear resolution of individual capillaries. A three-dimensional optoacoustic image enables detailed examination and analyses of tissue morphology and the vascular network. Optoacoustic micro-tomography with the fiber probe allows slices to be retrieved in different plane orientations, which can be stacked together to create volumetric images. Adapted from an image by the author (O. Ülgen) originally published in the thesis "Miniaturized Optical Ultrasound Detectors for High-Resolution Optoacoustic Imaging", Technical University of Munich, 2023 [1].

In-vivo imaging experiments

The effective use of our detector for *in-vivo* optoacoustic micro-tomography was validated by imaging back skin of athymic nude mouse. The *in-vivo* imaging experiments were conducted on an athymic nude mouse under general anesthesia (**Suppl. Fig.2d**). The vital signals of the mouse were simultaneously monitored and controlled throughout the experiments. Optoacoustic excitation and measurements were carried out through an opening at the bottom of a petri dish which was temporarily fixed on the back skin of the mouse. The fiber probe was placed close to the mouse skin during motorized scanning and signal acquisition. Limited-view optoacoustic micro-tomography experiments were performed by raster-scanning over the region of interest using high-speed motorized microscope translational stages (MLS203-1, Thorlabs) on which the samples were placed.

The micro-resonator's small size, combined with being positioned at the distal end of the optical fiber, allowed a short working distance from the sample. **Suppl. Fig.4** shows the maximum intensity projection **(Suppl. Fig.4b)** and depth-color-coded image **(Suppl. Fig.4c)** of the scanned region, with the scanned area outlined by a black frame in the bright-field reference image **(Suppl. Fig.4a)**. The detector exhibited high-resolution visualization of the microvasculature in the back skin of mouse, achieving remarkable clarity even at depths of up to 750 µm, which is beyond the capabilities of traditional optical microscopy methods. In addition, owing to its high sensitivity, the detector exhibited efficient performance at a modest fluence of around 2 mJ/cm^2, which is below the safe threshold for *in-vivo* procedures.

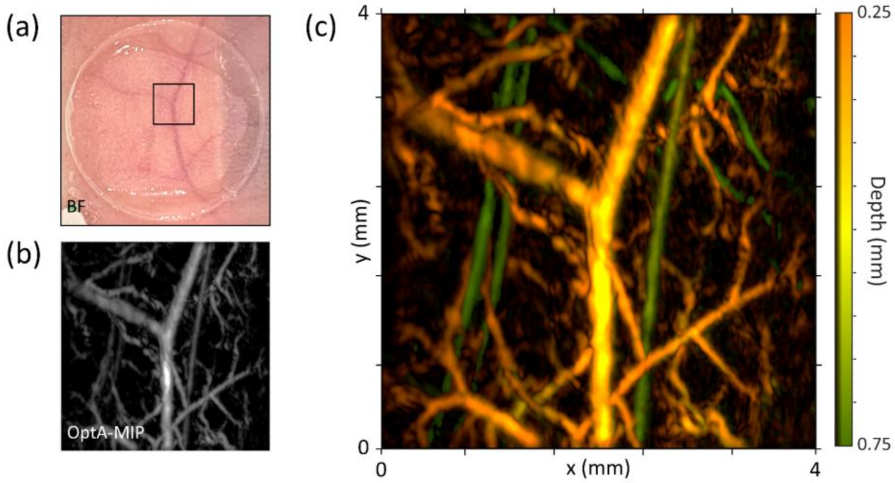

Supplementary Figure 4. In vivo optoacoustic imaging of mouse back skin. **(a)** A petri dish with a 20 mm opening is temporarily attached to the mouse skin. The region outlined on the bright-field image was raster-scanned with a 10 µm step size. **(b)** Maximum intensity projection (MIP) optoacoustic image was obtained with tomographic reconstruction. Areas of high intensity are shown in white, while those of low intensity are shown in black. **(c)** Depth-color-coded optoacoustic images encode depth information using color. The superficial regions are depicted in orange, while structures located deeper within the tissue are shown in green. The micro-vasculature is clearly resolved at distances up to 750 m using the fiber-based detector. During the experiment, the beam spot diameter was set to 1 mm and the fluence was less than 10 mJ/cm^2. Adapted from an image by the author (O. Ülgen) originally published in the thesis "Miniaturized Optical Ultrasound Detectors for High-Resolution Optoacoustic Imaging", Technical University of Munich, 2023 [1].

PR on tapered multi-core fiber

The conical distal end configuration was transferred to a multicore fiber to create a fiber probe that can deliver optoacoustic excitation light and detect generated ultrasound waves simultaneously **(Suppl Fig.5)**. In this design, the sensor readout was performed on the central core of the multi-core fiber, while the other cores, which were arranged in a hexagonal topology, were used to deliver the pulsed light for optoacoustic excitation. An axicon lens, formed by the fiber's conical distal end with a semi-vertical 30° angle, is a unique feature of this fiber probe. It produces a focal spot 470 µm from the fiber tip in pure water. In addition to allowing acoustic-resolution optoacoustic micro-tomography, the Bessel-like beam formed by the axicon lens allows for optical-resolution imaging. The optical focusing can be achieved without any additional front-end elements, and therefore affords a significant reduction in overall probe size.

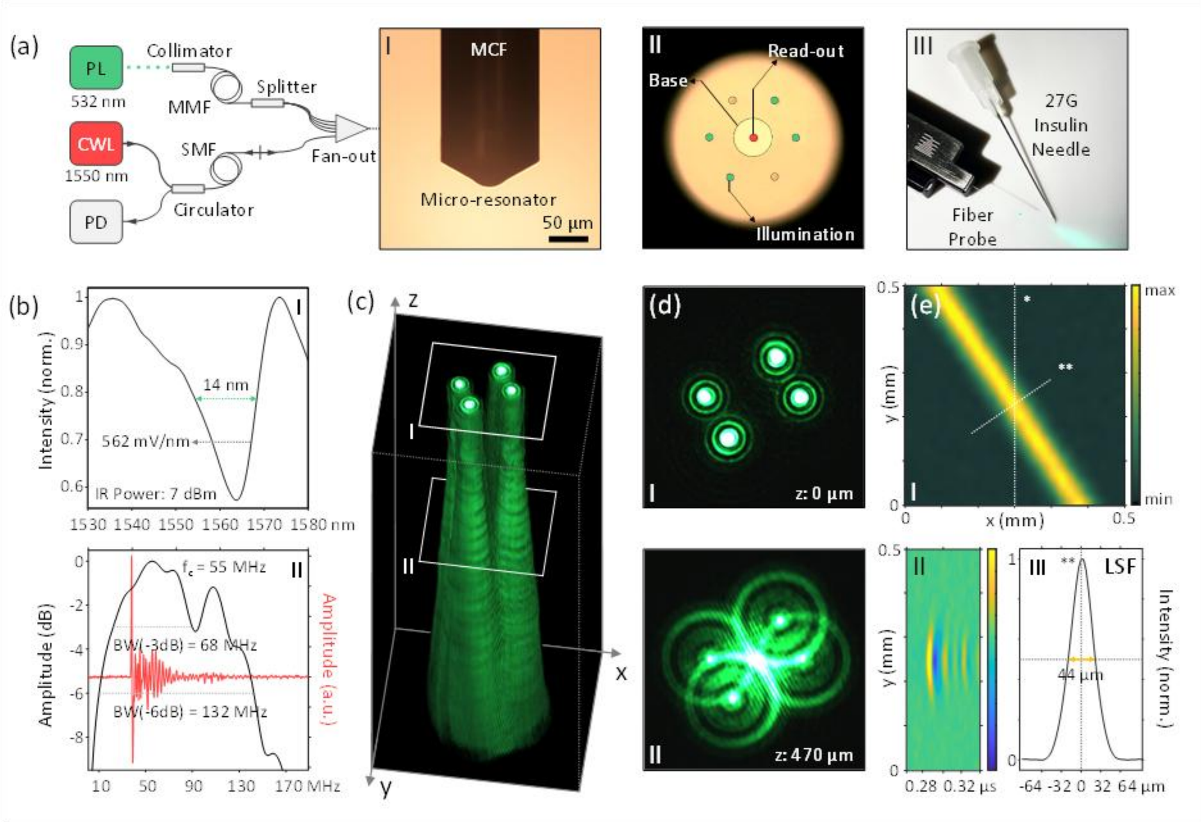

Supplementary Figure 5. Polymer resonator on multi-core tapered optical fiber. **(a)** Optical setup for coupling read-out and optoacoustic excitation beams into the multi-core fiber. **(I)** The side view of the multi-core fiber's distal end showing the polymer plano-concave cavity built on flat-cone shaped polished tip. **(II)** The cross-sectional top-view view of the multi-core fiber facet after polishing procedure. The single-mode core (8 µm) in the center serves as read-out channel (red) while the illumination channels (green) are in the circumference of the optical fiber. The remaining two cores were unused (yellow). The spacing between each 10-µm-diameter core is 30 µm, and the full diameter of the multi-core fiber is 125 µm. **(III)** The fiber probe has an extremely small size-factor and can fit into a 27G insulin needle. **(b) (I)** The reflection spectrum of the micro-resonator built at the fiber tip. The width of the resonance notch is measured to be 14 nm and off-resonance slope was 562 mV/nm. **(II)** The optoacoustic signal was acquired from a wideband ultrasound point source generated on a thin gold plate. The frequency response of the multi-core fiber detector shows its broadband response reaches 132 MHz at -6 dB and 68 MHz at -3 dB. The central frequency of the detector is 55 MHz. **(c)** The volumetric projection of the excitation beam from the multi-core fiber. **(d)** The cross-sections of the illumination field in the xy-plane at distances of 0 µm (I) and 470 µm (II). The conical tip of the fiber acts as an axicon lens and creates a Bessel-like beam. An optical focal spot with a focal length of 100 µm forms at a distance of 470 µm from the fiber tip. The focal spot allows optical-resolution optoacoustic imaging at sub-millimeter distances. At the same time, the detector is also capable of performing acoustic-resolution optoacoustic micro-tomography using the diffused light at higher distances. **(e)** Optoacoustic image of a 40-µm-diameter medical-grade polyamide suture embedded in agar phantom. **(II)** B-scan acquired along the line indicated by * in (e). **(III)** The intensity profile along the line indicated by ** in (e) and the line spread function showing that the diameter of the suture is measured to be 44 µm. Abbreviations: PL: Pulsed laser, CWL: Continuous-wave laser, PD: Photodiode, MMF: Multi-mode fiber, SMF: Single-mode fiber, MCF: Multi-core fiber, Norm: Normalized, BW: Bandwidth, LSF: Line spread function. Adapted from images by the author (O. Ülgen) originally published in the thesis "Miniaturized Optical Ultrasound Detectors for High-Resolution Optoacoustic Imaging", Technical University of Munich, 2023 [1].

References

1. Ulgen, O., Miniaturized Optical Ultrasound Detectors for High-Resolution Optoacoustic Imaging. PhD thesis, Technical University of Munich. (2023).
